# Moderate Intensity Intermittent Exercise Modality May Prevent Cardiovascular Drift

**DOI:** 10.3390/sports6030098

**Published:** 2018-09-15

**Authors:** Muzaffer Colakoglu, Ozgur Ozkaya, Gorkem Aybars Balci

**Affiliations:** Faculty of Sport Sciences, Ege University, 35100 Izmir, Turkey; ozgur.ozkaya@ege.edu.tr (O.O.); gorkem.aybars.balci@ege.edu.tr (G.A.B.)

**Keywords:** cardiac output, cardiovascular drift, interval training, stroke volume

## Abstract

Cardiovascular drift (CV-Drift) may occur after the ~10th min of submaximal continuous exercising. The purpose of this study was to examine whether CV-Drift is prevented by an intermittent exercise modality, instead of a continuous exercise. Seven well-trained male cyclists volunteered to take part in the study ($\dot{V}$O_2max_: 61.7 ± 6.13 mL·min^−1^·kg^−1^). Following familiarization sessions, athletes’ individual maximal O_2_ consumption ($\dot{V}$O_2max_), maximum stroke volume responses (SV_max_), and cardiac outputs (Qc) were evaluated by a nitrous-oxide re-breathing system and its gas analyzer. Then, continuous exercises were performed 30 min at cyclists’ 60% $\dot{V}$O_2*max*_, while intermittent exercises consisted of three 10 min with 1:0.5 workout/recovery ratios at the same intensity. Qc measurements were taken at the 5th, 9th, 12nd, 15th, 20th, 25th, and 30th min of continuous exercises versus 5th and 10th min of workout phases of intermittent exercise modality. Greater than a 5% SV decrement, with accompanying HR, increase, while Qc remained stable and was accepted as CV-Drift criterion. It was demonstrated that there were greater SV responses throughout intermittent exercises when compared to continuous exercises (138.9 ± 17.9 vs. 144.5 ± 14.6 mL, respectively; *p* ≤ 0.05) and less HR responses (140.1 ± 14.8 vs. 135.2 ± 11.6 bpm, respectively; *p* ≤ 0.05), while mean Qc responses were similar (19.4 ± 2.1 vs. 19.4 ± 1.5 L, respectively; *p* > 0.05). Moreover, the mean times spent at peak SV scores of exercise sessions were greater during intermittent exercise (1.5 vs. 10 min) (*p* < 0.001). In conclusion, intermittent exercises reduce CV-Drift risk and increases cardiac adaptation potentials of exercises with less physiological stress.

## 1. Introduction

Cardiovascular drift (CV-Drift) is characterized by a gradual decrease in stroke volume (SV) and an increase in heart rate (HR), whereas cardiac output (Qc) remains stable without an increase in exercise intensity. CV-Drift usually appears after 10th min of constant load prolonged heavy and very heavy exercises [1,2]. These intensities corresponded between aerobic threshold and slightly above the anaerobic threshold [2,3].

In the traditional concept, CV-Drift occurs due to SV decrement in warm environmental conditions based on a progressive increase in cutaneous blood flow, as body temperature and dehydration rise [4]. Alternatively, drift reveals by a second HR increase after an exercise-induce HR increment based on catecholamine rise in thermo-neutral conditions [4,5]. If exercise duration is several hours and noticeable dehydration becomes apparent, cardiac output would also decline even in a thermo-neutral environment [6]. On the other hand, drift-time and their depths are closely related with the exercise intensity, duration, modality, and activated muscle proportion, etc. [7,8,9].

The WHO has recommended prolonged (>60-min) conservative brisk walking or running at the intensity corresponding to 6–10 MET (50–60% of predicted $\dot{V}$O_2*max*_) [10]. Moreover, long-term prolonged exercises have been traditionally used as training modalities to improve aerobic performance for especially long distance endurance athletes. However, it has been reported that SV responses can decrease up to 20% after the 10th–15th min of the prolonged exercises [11]. Since time spent at maximum SV levels (*t*→*SV_max_*) is one of the most important training stimuli [12,13,14], potential $\dot{V}$O_2*max*_ development would be adversely affected if CV-Drift occurs during a prolonged exercise. Furthermore, SV*_max_* responses of non-elite athletes have usually corresponded to lower fractional usages (40–80% of $\dot{V}$O_2*max*_) of their individual $\dot{V}$O_2*max*_ levels [15]. Therefore, exercise intensities corresponding to SV*_max_* responses, instead of $\dot{V}$O_2*max*_, may be a better exercise stimuli for aerobic power and capacity development [16]. In this case, preventing CV-Drift may provide more suitable physiologic conditions for both SV*_max_* and therefore $\dot{V}$O_2*max*_ development.

The hypothesis of this study is, instead of a traditional continuous exercise, an intermittent exercise modality, which is a better training strategy to prevent CV-Drift by increasing SV responses of training session and *t*→*SV_max_*. The purpose of this study was therefore to analyses Qc, SV, HR, and $\dot{V}$O_2_ responses as well as rates of perceived exertion (RPE) throughout the submaximal continuous and intermittent exercise modalities.

## 2. Materials and Methods

The study was designed according to rules and principles of the Helsinki Declaration and was approved by the university ethics committee (CBU.ETK.20478486-84). Informed consent in writing was obtained after explaining the nature of the study, risks and benefits of participating in this study.

### 2.1. Participants

Seven well-trained male cyclists volunteered to take part in this study (age: 22 ± 2.4 years; body mass: 71.7 ± 9.3 kg; height: 178.1 ± 6 cm; body fat: 8.8 ± 1.9%; $\dot{V}$O_2*max*_: 62.6 ± 6.8 mL·min^−1^·kg^−1^). At the time of the study, participants were involved in 5.3 ± 1.3 training sessions per week. Their average athletic experience was 7.4 ± 2.8 years. All procedures were performed using standard conditions of 20–21 °C temperature and 50–55% relative humidity in a climatic chamber. The study was conducted after the competition season ended in order to minimize training effects or periodization, and was completed within 14 days. In addition, testing time of the day was standardized to minimize any effect of circadian variance for each volunteer. Since fluid intake may affect drift phenomena, participants consumed approximately 300 mL of water one hour before their experiments. They were requested not to take part in any exhaustive exercise and did not consume any caffeine-containing drink or food in the period of the study. Moreover, none of the participants were not taking any medication or nutritional supplements.

### 2.2. Experimental Design

**Figure 1:**
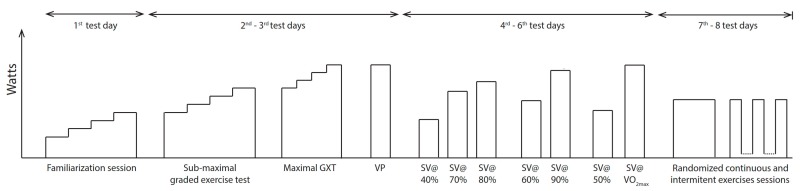
Flow chart of experimental process.

A crossover study design was used for this laboratory assessment. Firstly, familiarization sessions were performed to acclimate participants to electromagnetically braked cycle ergometer, climatic chamber conditions and our research group. Following familiarization sessions, a submaximal incremental test was performed to determine athletes’ ventilatory threshold. Then, a maximal incremental test was conducted from ventilatory threshold to volitional exhaustion within 12 min by using approximately ~30 watt increments grade-by-grade. The highest 30 s average of $\dot{V}$O_2_ (mL·min^−1^·kg^−1^) throughout the test was calculated as the $\dot{V}$O_2*peak*_. On the subsequent day, a verification phase to volitional exhaustion was conducted by using a constant workload that was equaled to the power output corresponding to $\dot{V}$O_2*peak*_. The highest 30 s average of $\dot{V}$O_2_ throughout the verification phases were accepted as the $\dot{V}$O_2*max*_ [17]. Moreover, workloads ranged from 40% and 100% of $\dot{V}$O_2*max*_ were analyzed to reveal athletes’ individual SV*_max_* responses, and its workload, by using constant load exercises. SV measurements were taken by nitrous-oxide re-breathing (N_2_O_RB_) method. Continuous exercises were performed at 60% of $\dot{V}$O_2*max*_ for 30 min, while intermittent exercises were conducted as three 10-min workloads with the 5-min rests by using the same intensities and workout durations. Hypotheses were then tested by means of $\dot{V}$O_2_ (mL·min^−1^·kg^−1^), Qc (L·min^−1^), SV (mL), HR (bpm), arterio-venous oxygen differences (a-vO_2difference_) (%), respiratory exchange ratio (RER), and RPE. Additionally, *t*→*SV_max_* was calculated as elapsed time in minutes by the criterion as close as 5% to SV*_max_*. It was assumed that SV has linear trend between time points of N_2_O_RB_ measurements during intermittent exercise. A flow chart is shown in Figure 1.

### 2.3. Procedures

#### 2.3.1. Cycle Ergometer

In this study, a computer-controlled electromagnetically braked cycle ergometer with hyperbolic mode that allows fixing external power output at self-cadence (Lode BV, Excalibur Sport, Lode Medical Technology, Groningen, The Netherlands) was used. The seat and handlebar heights were adjusted for each participant to allow a slight bend on the knee when their foot was at the lowest position on the pedal.

#### 2.3.2. $\dot{V}$O_2max_ Determination

HR, breath-by-breath, $\dot{V}$O_2_ and $\dot{V}$CO_2_ were measured by a gas analyzer (Innocor Inno-0500, Innovision A/S, Odense, Denmark). Sessions consisted of two successive visits. In the first session, experiments were simulated with participants. On the following day, a submaximal incremental test consisting of four 5-min stages was performed by using ~25–30 watt increments for each stage. The test continued until reaching approximately 50% of maximal heart rate reserve. After familiarization sessions, submaximal incremental tests consisted of at least four 5-min stages. The initial workload was set at 50–60% of the heart rate reserve. At each stage, the workload was increased by ~30 watts. The procedure continued till the ventilatory threshold was revealed by the V-slope method [18]. The ventilatory threshold obtained from the submaximal incremental test was used to adjust the initial workload of maximal incremental aerobic power tests [19]. Aerobic power tests consisted of 2-min stages to volitional exhaustion. Workload was increased by ~30 watts for each stage. Participants received verbal encouragements, especially after the first stage, in order to produce maximal effort. When the maximal incremental test was terminated by athletes’ volitional exhaustion, the second control criteria were used to check athletes’ fatigue conditions by (i) a plateau in VO_2_ (VO_2_ difference less than 150 mL·min^−1^); (ii) greater than 90% of age-predicted maximum (220-age) HR (beats·min^−1^); (iii) greater than 1.1 or above respiratory exchange ratio (RER) [20] and (iv) rate of the perceived exertion (RPE) of 19–20 in Borg’s 15-point scale [21]. $\dot{V}$O_2*peak*_ was determined as the highest 30 s average of $\dot{V}$O_2_ throughout the test. If the elapsed time for the maximal incremental test was not within the 12 min, the test was repeated another day to obtain standardization for determining $\dot{V}$O_2*peak*_ [22]. Constant-load verification phases were then conducted to verify the results of the maximal incremental test. Constant load verification phases were performed with the verbal encouragements throughout the test until reaching to the limits of tolerance and workload was set at the wattage to reveal the highest 30 s average $\dot{V}$O_2_ derived from maximal incremental test. Verification procedure termination criteria were accepted as the same with maximal incremental tests’. The highest 30 s average of $\dot{V}$O_2_ throughout the test was accepted as verified $\dot{V}$O_2*max*_.

#### 2.3.3. SV_max_ Determination

After $\dot{V}$O_2*max*_ determination, constant-load SV*_max_* detection procedure was conducted to reveal individuals’ SV*_max_* responses. A valid and reliable noninvasive inert-gas N_2_O_RB_ was used for Qc measurements (Innocor INN00500, Innovision A/S, Odense, Denmark) [23]. The workloads from 40 to 70% of $\dot{V}$O_2*max*_ were maintained for 10 min, in the meantime, three N_2_O_RB_ were completed between 4:30–5:00, 7:00–7:30 and 9:30–10:00 min. In addition to that, workloads in the range of 80–100% of $\dot{V}$O_2*max*_ were sustained for 5 min, and N_2_O_RB_ were accomplished between 2:30–3:00, 4:30–5:00, and 6:30–7:00 min, if the test was prolonged. SV was calculated by dividing Qc by the HR. HR was recorded via the pulse-oximeter of the system and an external heart rate monitor (Polar RS400; Polar Electro Oy, Kempele, Finland). Device calibrations were undertaken according to the manufacturer’s instructions. If the N_2_O increased at the end of the test due to the recirculation, the measurement was cancelled and repeated on a subsequent day. At the beginning of each measurement, end-tidal gas values were checked whether N_2_O and SF_6_ were above 0.002% and 0.001%, respectively. Breath-by-breath $\dot{V}$O_2_ and $\dot{V}$CO_2_ were measured using the same N_2_O_RB_ system.

#### 2.3.4. Continuous and Intermittent Exercise Bouts

Continuous and intermittent exercises were performed with 24–48 h intervals. Exercise intensities were fixed at the wattage corresponding to 60% of individuals’ $\dot{V}$O_2*max*_ values (30-min continuous exercise vs. three 10-min intervals with 5-min passive recovery by using crossover experimental design). A pilot study was performed with three participants to decide intermittent exercises duration. Five, 10, and 15-min intervals were examined with 1:2 or 1:1 rest to work ratio. Therefore, the duration of intermittent exercise was set to be short enough to be affected by CV-drift.

SV data was not recorded continuously during the tests since the N_2_O_RB_ method needs sufficient time for the washout of inert gasses (N_2_O and SF6) between repeated measurements for the same individual. The time delay of approximately 5 min at rest and 2–3 min during high to moderate intensity exercise for subsequent measurements was necessary. Therefore, N_2_O_RB_ was applied at the 5th, 9th, 12nd, 15th, 20th, 25th, and 30th min for continuous exercises, while it was applied at 5th and 10th min of workout phases for intermittent exercise modality. As regards to 5th min of measurement, subsequently measured SV, HR and Qc data were evaluated; it was considered as drift criterion that there is a greater than 5% decrease in SV whereas Qc is maintained nearly constant throughout exercises.

### 2.4. Statistical Analysis

Results were evaluated using SPSS 20.0 (SPSS Inc., Chicago, IL, USA) statistical software. Descriptive results were reported as mean values and standard deviations (±SD). After Skewness and Kurtosis, Shapiro-Wilk test of normality was used to determine whether the distribution of values is normal or not. Paired samples *t*-test was then conducted to assess differences in $\dot{V}$O_2_ (mL·min^−1^·kg^−1^), Qc (L·min^−1^), SV (mL), *t*→*SV_max_*, HR (bpm), a-vO_2difference_ (%), RER (VCO_2_/VO_2_) and RPE (6 to 20 Borg scale) means. a *p* ≤ 0.05 were considered as statistically significant.

## 3. Results

Main results showed that a clear CV-Drift characterized by progressively decrease in SV and parallel increase in HR, whereas Qc was maintained nearly constant and occurred during continuous exercises for all participants. After 12th min measurements, SV responses began to progressively decrease, while HR continued its increase (*p* ≤ 0.05). On the other hand, throughout the intermittent exercises, SV responses were greater and therefore both Qc and VO_2_ means were higher (Table 1).

**Figure 2:**
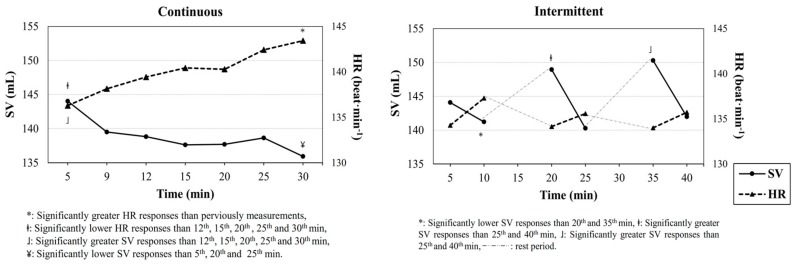
Stroke volume and heart rate responses during intermittent and continuous exercises.

Moreover, *t*→*SV_max_* scores obtained from intermittent exercises (10 min) were greater than that of continuous exercise modality (1.5 min). Time depended SV and HR responses are shown in Figure 2.

## 4. Discussion

The main outcomes of this study may be reviewed as (i) there was a greater CV-Drift pattern observed in continuous exercises, however CV-Drift effects minimized by intermittent exercise modality; (ii) intermittent exercises provided greater SV responses throughout the workload periods (~145 mL) compared to continuous exercises (~140 mL); (iii) there were greater *t*→*SV_max_* scores during the intermittent exercise modality versus continuous exercise (10 vs. 1.5 min, respectively).

SV improvement is a key factor of $\dot{V}$O_2*max*_ development, because SV, instead of HR or a-vO_2difference_, is known to be the main predictor of aerobic fitness [15]. Higher SV responses during exercises can result in a positive effect for heart tissue. Therefore, it should be taken into consideration, this knowledge, when deciding on the exercise model. However, World Health Organization (WHO) has recommended continuous-type brisk walking or running at moderate intensities (≥6 MET per session; ≥3 sessions per week; ≥60–90 min per week) to optimize cardiorespiratory functions and to prevent chronic diseases. Though, CV-Drift occurs especially at this safe mode of continuous exercises, corresponding to moderate intensities as a result of that SV level, which can be decreased dramatically throughout the exercise period. In this study it was showed that SV responses tend to decrease during the continuous exercise period. This can negatively affect aerobic fitness enhancement due to lower adaptation in the heart tissue. Additionally, it is a generally accepted view that CV-Drift occurs due to a progressive increase in cutaneous blood flow, as the body temperature rises. The rise in cutaneous blood flow is thought to lead to an increase in cutaneous venous volume, thus reducing ventricular filling pressure, end-diastolic volume, and SV during moderate-intensity exercise [4]. Accordingly, CV-Drift may negatively affect training efficiency by diminishing cardiac performance. Those negative effects can be minimized by using recovery intervals, instead of continuous, during moderate intensity exercises. Beside the health benefits, performance-focused athlete groups should also use intermittent exercise modalities versus continuous exercises performed at moderate intensities. In this study it was showed that CV-Drift is minimized by using 5-min rest in every 10-min of exercise (Figure 2). Thus, discontinuous exercises may be a good exercise strategy to improve aerobic fitness, since it minimizes or prevents CV-Drift. On the other hand, due to the interval specificity of discontinuous training modality, there was less physiological stress in intermittent exercise sessions compared to continuous exercises.

It is known that one of the most important acute training effects is the amount of t→*SV_max_* throughout exercise sessions [13]. In present study, there was a greater t→*SV_max_* score obtained from intermittent exercises versus the continuous exercise modality. t→*SV_max_* was 10 min in intermittent exercises, while it was just 1.5 min for traditional continuous exercises. For this reason, intermittent exercises can be lead to improve aerobic fitness and cardiac health.

Additionally, CV-Drift had previously tried to be prevented by methods such as invasive glucose infusion [6] or a small dose of beta-blocker intake (i.e., Atenolol) [5]. However, these non-practical and invasive interventions have posed risks for professionals and people who have circulatory abnormalities. On the other hand, CV-Drift risks may only be prevented by changing the exercise modality without any invasive interventions. Instead of a continuous exercise model, intermittent exercises may effectually minimize the risks of CV-Drift by increasing SV responses throughout the exercise session leading to the t→*SV_max_* being greater. It is shown that in each repetition of intermittent exercises, SV responses tend to decrease in the 10th min. This result showed that moderate intensity intermittent exercises repetitions should not be longer than 10 min to maintain higher SV. These findings may improve cardiac adaptation to exercise as well as to make a significant contribution for preventive medicine and cardiac rehabilitation areas, in addition to athletic performance.

## 5. Conclusions

It has been shown that CV-Drift was prevented by various methods, being glucose infusion [6], a small dose of Atenolol intake [5], or very high volume exercise lasting nearly 12 h [24]. However, according to our knowledge, this is the first study to show that aerobic intervals may prevent CV-Drift by increasing SV responses and *t*→*SV_max_* throughout the exercise session. For this reason, intermittent modalities may improve cardiac adaptation better than a continuous one. In addition, due to the interval specificity of the discontinuous training modality, there is less physiological stress in intermittent exercise sessions compared to continuous exercises. Future studies should be focus on untrained participants. Similar results could be expected for them. These findings are important for both athletic performance and preventive medicine or cardiac rehabilitation areas. On the other hand, a limitation for this study is the time delay resulting from emptying and refilling the rebreathing bag for new measurement, which takes approximately two minutes to avoid recirculation. Thus, a minimum of two-to three-minute intervals were necessary between Qc measurements.

## Figures and Tables

**Table 1:** Tested variables obtained from both continuous and intermittent exercise modalities. Data are means ± SD.

Variables	Continuous	Intermittent	*t*	*p*	Cohen’s *d*
HR (beat · min^−1^)	140.1 (14.8)	135.2 (11.6)	3.414	0.014	0.38
SV (mL)	138.9 (17.9)	144.5 (14.6)	−2.860	0.029	−0.34
*t*→*SV_max_* (min)	1.5 (0.5)	10 (2)	9.245	0.000	−5.83
Qc (L)	19.3 (2.1)	19.4 (1.5)	−0.393	0.708	−0.32
a-vO_2difference_ (%)	13.7 (1.6)	13.2 (1)	17.441	0.000	−0.05
$\dot{V}$O_2_ (mL · min^−1^ · kg^−1^)	37.3 (2.8)	36.2 (2.6)	2.836	0.30	0.41
RER	0.86 (0.04)	0.85 (0.06)	1.362	0.222	0.20
RPE	11.1 (1.3)	11 (1.8)	0.420	0.689	0.06

HR. Heart rate; SV: Stroke volume; Qc: Cardiac output; t→*SV_max_*: Time spent at maximal stroke volume based on as close as 5% response to real maximum; a-vO_2difference_: Arterio-venous oxygen difference; $\dot{V}$O_2_: Oxygen consumption value per body mass; RER: Respiratory exchange ratio; and RPE: Rate of perceived exertion.
